# UWB/Binocular VO Fusion Algorithm Based on Adaptive Kalman Filter

**DOI:** 10.3390/s19184044

**Published:** 2019-09-19

**Authors:** Qingxi Zeng, Dehui Liu, Chade Lv

**Affiliations:** 1 College of Automation Engineering, Nanjing University of Aeronautics and Astronautics, Nanjing 211106, China; 2 Science and Technology on Communication Networks Laboratory, Shijiazhuang 050000, China; 3 Nondestructive Detection and Monitoring Technology for High Speed Transportation Facilities, Key Laboratory of Ministry of Industry and Information Technology, Nanjing 210000, China

**Keywords:** ultra-wideband (UWB), binocular VO, sensor fusion, adaptive kalman filter

## Abstract

Among the existing wireless indoor positioning systems, UWB (ultra-wideband) is one of the most promising solutions. However, the single UWB positioning system is affected by factors such as non-line of sight and multipath, and the navigation accuracy will decrease. In order to make up for the shortcomings of a single UWB positioning system, this paper proposes a scheme based on binocular VO (visual odometer) and UWB sensor fusion. In this paper, the original distance measurement data of UWB and the position information of binocular VO are merged by adaptive Kalman filter, and the structural design of the fusion system and the realization of the fusion algorithm are elaborated. The experimental results show that compared with a single positioning system, the proposed data fusion method can significantly improve the positioning accuracy.

## 1. Introduction

In an indoor environment, obtaining the location and orientation of a moving vehicle is an important part of the autonomous navigation of indoor vehicles. In some occasions where high indoor positioning accuracy is required, a high-precision optical motion capture system [1] can be selected, which can provide centimeter-level positioning accuracy. However, it is costly and complicated in structure, and has high requirements for layout and installation in an indoor scene, so it cannot be widely used in the field of vehicle interior positioning. The inertial navigation system (INS) can provide positioning information for the vehicle both indoors and outdoors, but its positioning accuracy is directly proportional to the manufacturing cost. For the inertial navigation system used in the vehicle, its positioning error will accumulate over time, and it cannot provide long-term reliable positioning for the vehicle [2]. With the development of digital image processing technology and advances in the fields of high-performance processors and CCD (charge-coupled device) sensors, vision-based odometer technology has gradually become a research hotspot in the field of navigation. The visual odometer estimates the pose of the camera by analyzing and processing the sequence of images between adjacent frames. Similar to the IMU (inertial measurement unit), its positioning error will also drift, which is not suitable for long-term positioning navigation [3].

In recent years, wireless indoor positioning systems have become more and more popular, and IPS (indoor positioning systems) can provide global positioning information for indoor mobile vehicles. Existing wireless positioning systems include RFID (radio frequency identification) [4], WIFI [5], ZigBee [6], and so on. Although RFID is low in cost and compact in structure, it is less affected by non-line of sight [7], however, it has a small scope of work, does not have communication capabilities, and requires a large amount of infrastructure to accurately locate. WIFI is easily interfered by mobile devices, resulting in poor positioning accuracy, so it is not suitable for indoor positioning of vehicles. In the indoor environment, ZigBee has low positioning accuracy due to the influence of the building structure [8]. Compared with the above wireless positioning scheme, ultra-wideband (UWB) [9] is one of the most promising indoor positioning technologies. UWB has the characteristics of low power consumption and high bandwidth, and can transmit a large amount of data with low energy consumption. At the same time, UWB has strong penetrating power, which can reduce multipath interference and provide higher positioning accuracy [10]. Bastida-Castillo et al. used UWB technology to track the position of athletes and compare them with GPS (global positioning system) technology. Experiments show that UWB is more accurate than GPS in determining distance and measuring average speed [11]. In contrast, UWB can not only accurately estimate the player’s position on the court, but also because of the automatic and portable features of UWB, it is very suitable for tactical analysis and measurement [12]. Bastida-Castillo et al. also verified that the UWB positioning system has strong reliability, and the use of different devices does not have a significant impact on measurement error [13]. In order to feedback potential dangers to road users, especially cyclists, Davide et al. [14] proposed a positioning system based on UWB technology. Bicycles were equipped with low-cost, low-complexity active tags and HMIs (human machine interfaces), and reference nodes were placed at intersections (infrastructure-based architecture) or vehicles (vehicle-based architecture). The results show that even in the harsh propagation environment, sub-meter positioning accuracy and good tracking ability can be achieved. Considering that UWB has the above characteristics, this paper chooses the UWB-based fusion system as the indoor positioning scheme for mobile vehicles.

## 2. Related Work

If the UWB positioning system wants to achieve high-precision positioning performance, it needs to work in a limited indoor scene. However, in practical applications, the indoor environment is affected by complex factors such as building structure and scene distribution. UWB faces problems such as non-line of sight (NLOS) and multipath, which causes the positioning accuracy of the UWB system to be impaired. In order to overcome the shortcomings of the single UWB positioning method, relevant literature based on UWB multi-sensor fusion has emerged to improve the accuracy of navigation and positioning.

The existing UWB integration scheme generally considers the use of an inertial navigation system (INS) to meet the positioning requirements by blending data information from the INS. Hol et al. [15] proposed a tight coupling method based on UWB and inertial sensors to estimate the position and attitude of 6DOF (degree of freedom). They used accelerometers, gyroscopes, and time of arrival (TOA) measurements directly for sensor fusion, rather than the filtered position or acceleration. Experiments show that even with multipath and non-line of sight, the tightly coupled system can still obtain robust and accurate positioning results. However, the experimental mileage is shorter and the positioning accuracy in height is poor. In order to solve the delay and low bandwidth problem caused by the UWB constant speed hypothesis, Li et al. [16] proposed an Extended Kalman Filter (EKF)-based algorithm to fuse the inertial measurement unit (IMU) and UWB, in which the state vector of EKF takes into account the acceleration deviation in three directions. Experiments showed that this method can significantly improve the positioning accuracy and reduce the delay. However, in practical applications, it is difficult to obtain accurate IMU acceleration deviation, and when the MAV (Micro Air Vehicle) is landing, the IMU measurement will produce an abnormality, and the positioning performance of the fusion system relying on the acceleration information will be significantly reduced. Benini et al. [17] used the UbiSense UWB real-time positioning system and the low-cost IMU to perform the fusion by the Biased Extended Kalman Filter (BEKF). In order to improve the positional accuracy, the deviation and scale factor of the accelerometer and the gyroscope were also considered. However, the fusion algorithm does not use the original measurement data of UWB, but uses the estimated three-dimensional coordinates (x, y, z) of the UWB system as the observation of the Kalman filter. In order to overcome the shortcomings of UWB and IMU, Yao et al. [18] merged the two by extending the Kalman filter (EKF). The UWB was used to calibrate or estimate the IMU error, while the IMU was used to detect and isolate the damaged UWB sensor data to improve overall positioning performance.

In recent years, applications that use monocular or stereoscopic information to blend other sensors to improve positioning have become more common. Among them, visual aid UWB was used to improve the estimation accuracy of helicopter landing missions [19], Hoeller et al. integrated sensor measurement information to learn and compensate for the deviation of the UWB positioning system. In the case of visual impairments, this compensation bias could be used to improve the accuracy of the task. However, the article uses a visual method based on planar artificial markers, and the vision is mainly used to assist UWB to improve the estimation accuracy of helicopter landing missions. Tiemann et al. [20] used the data of the monocular SLAM (Simultaneous Localization and Mapping) system to enhance UWB positioning performance. Since the position estimation of the monocular SLAM has no global reference information and scale factor, the article used UWB position information to estimate and optimize those unknown parameters to achieve automatic flight of the UAV (Unmanned Aerial Vehicle) in the area where the wireless positioning was not covered. However, the method proposed in the article needs to be equipped with a highly networked architecture, and frequent information exchange between systems is required. Benini et al. [21] fused low-cost IMU, UWB, and visual information through EKF, but the visual odometer is used to detect the marked pictures near the UAV proximity area, and the fusion method is mainly used to improve the local positioning effect of the micro drone. Due to the scale uncertainty of the monocular visual odometer, it is impossible to provide an accurate global reference position for indoor autonomous navigation [20]. Compared to monocular, the binocular scheme can calculate depth information to provide more accurate positioning results [22]. Therefore, the main work of this paper is to use the position information of the binocular visual odometer (VO) to integrate with UWB to improve the positioning accuracy of UWB indoors.

The structure of this paper is as follows: Section 3 introduces the principle of UWB ranging and positioning. Section 4 first introduces the depth calculation principle of the binocular camera, and then briefly describes the feature-based binocular visual mileage calculation method. Section 5 describes the structural design of the fusion system of UWB and binocular VO, and then describes the modeling process of the fusion algorithm. Section 6 introduces the configuration of the experimental hardware and the construction of the experimental platform, then analyzes the experimental results. Section 7 summarizes the fusion algorithm proposed in this paper.

## 3. UWB (Ultra-Wideband) Positioning System

The UWB positioning system consists of a tag and several anchor sensors. The process of obtaining the three-dimensional coordinates of the tag sensor generally consists of two parts: (1) use the TOF (time of flight) ranging method to obtain the distance between the tag and anchors, and (2) calculate the coordinates of the tag using the trilateration method [23] or the least squares method [24] based on the measured distance.

### 3.1. UWB Ranging Principle

The TOF ranging method belongs to the two-way ranging technology, which mainly uses the propagation time of signals between two asynchronous receivers to measure the distance between nodes. Since the distance obtained by the TOF ranging technology is linear with the signal propagation time, the distance measurement result of the method is more accurate in an ideal viewing environment.

**Figure 1:**
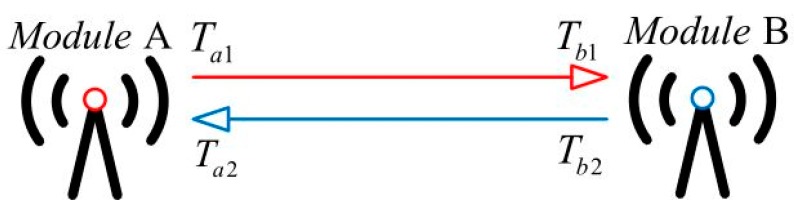
Two way-time of flight ranging principle.

The ranging method of the UWB module used in this paper is the two way-time of flight (TW-TOF), and its ranging principle is shown in Figure 1. When two modules are started, each module generates an independent timestamp. Module A’s transmitter transmits a requested pulse signal to module B on its timestamp T*_a_*_1_, then module B receives the signal at its own timestamp T*_b_*_1_. Module B in turn transmits a response signal to module A at a time T*_b_*_2_, and module A receives the signal at a time T*_a_*_2_. According to the propagation time of the pulse signal between the two modules, the distance *D* between the two modules can be calculated by Equation (1), where *C* represents the speed of light.

(1) $$D=C*\left[\left(T_{a2}-T_{a1}\right)-\left(T_{b2}-T_{b1}\right)\right]/2$$

### 3.2. UWB Positioning Principle

**Figure 2:**
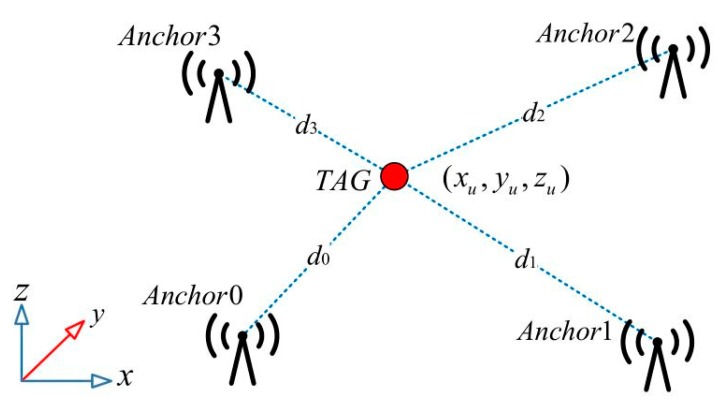
Positional relationship between the anchors and the tag.

In order to obtain the three-dimensional coordinates of the tag, this paper requires 4 anchor sensors to be used together. According to the positioning relationship of the anchors and the label shown in Figure 2, the distance Formula (2) is as follows:

(2) $$\begin{array}{l} \sqrt{{\left(x_{0}-x_{u}\right)}^{2}+{\left(y_{0}-y_{u}\right)}^{2}+{\left(z_{0}-z_{u}\right)}^{2}}=d_{0}\\ \sqrt{{\left(x_{1}-x_{u}\right)}^{2}+{\left(y_{1}-y_{u}\right)}^{2}+{\left(z_{1}-z_{u}\right)}^{2}}=d_{1}\\ \sqrt{{\left(x_{2}-x_{u}\right)}^{2}+{\left(y_{2}-y_{u}\right)}^{2}+{\left(z_{2}-z_{u}\right)}^{2}}=d_{2}\\ \sqrt{{\left(x_{3}-x_{u}\right)}^{2}+{\left(y_{3}-y_{u}\right)}^{2}+{\left(z_{3}-z_{u}\right)}^{2}}=d_{3} \end{array}$$

where, $x_{i},y_{i},z_{i}(i=0,1,2,3)$ represents the known position of the 4 anchor points in the coordinate system, and $\left(x_{u},y_{u},z_{u}\right)$ represents the position of the label in the coordinate system, the position of which is unknown.

It can be seen from the above formula that although there are four equations in Equation (2), there are only three unknowns, and an accurate solution cannot be obtained. Therefore, we can use the iterative method and the least squares method to estimate the optimal solution $\left(\hat{x},\hat{y},\hat{z}\right)$ of the tag position.

(3) $$(\hat{x},\hat{y},\hat{z})=\underset{\left(x_{u},y_{u},z_{u}\right)}{\min}{\sum_{i=0}^{3}\left[d_{i}-\sqrt{\left(x_{i}-x_{u})+(y_{i}-y_{u})+(z_{i}-z_{u}\right)}\right]}^{2}$$

Since Equation (2) is a nonlinear system of equations, the common solution is to linearize it. The above Equation (3), can be simplified to Equation (4), where *i* = 0, 1, 2, 3:

(4) $$d_{i}=\sqrt{{\left(x_{i}-x_{u}\right)}^{2}+{\left(y_{i}-y_{u}\right)}^{2}+{\left(z_{i}-z_{u}\right)}^{2}}$$

Linearize this equation and get the result as Equation (5):

(5) Δd=HΔx

where, Δd and Δx are vectors and *H* is a matrix, which respectively represent the following meanings:

(6) $$\Delta d={\left[\begin{array}{cccc} \delta d_{0} & \delta d_{1} & \delta d_{2} & \delta d_{3} \end{array}\right]}^{T}$$

(7) $$\Delta x={\left[\begin{array}{ccc} \delta x_{u} & \delta y_{u} & \delta z_{u} \end{array}\right]}^{T}$$

(8) $$H=\left[\begin{array}{ccc} \frac{x_{0}-x_{u}}{d_{0}} & \frac{y_{0}-y_{u}}{d_{0}} & \frac{z_{0}-z_{u}}{d_{0}}\\ \frac{x_{1}-x_{u}}{d_{1}} & \frac{y_{1}-y_{u}}{d_{1}} & \frac{z_{1}-z_{u}}{d_{1}}\\ \frac{x_{2}-x_{u}}{d_{2}} & \frac{y_{2}-y_{u}}{d_{2}} & \frac{z_{2}-z_{u}}{d_{2}}\\ \frac{x_{3}-x_{u}}{d_{3}} & \frac{y_{3}-y_{u}}{d_{3}} & \frac{z_{3}-z_{u}}{d_{3}} \end{array}\right]$$

Since *H* is not a square matrix, its inverse matrix cannot be directly solved. In Equation (5), there are four equations in the linear equations, more than the unknowns, which can be solved by the least squares method. By solving the pseudo inverse matrix for *H*, the solution of the system of equations can be obtained:

(9) $$\delta x={\left[H^{T}H\right]}^{-1}H^{T}\delta d$$

## 4. Visual Odometer (VO)

In the field of computer vision and mobile robotics, vision-based odometer technology has been widely used. The technique can obtain the 6-degree-of-freedom motion (position, attitude) of the camera by analyzing and processing the sequence of adjacent frame images, and restore the spatial structure of the scene. Visual odometers typically use a monocular camera or a binocular camera solution. Since the image acquired by the monocular camera lacks depth information, there is an uncertain scale factor for the motion trajectory of the monocular visual odometer. Compared to the monocular visual odometer, the stereo vision odometer can recover the depth information by the sequence of images acquired by the left and right cameras, so the stereo vision odometer can estimate the exact trajectory. Therefore, the camera used in this article is a binocular RGB (red, green, blue) camera.

### 4.1. Binocular Camera Depth Calculation Principle

**Figure 3:**
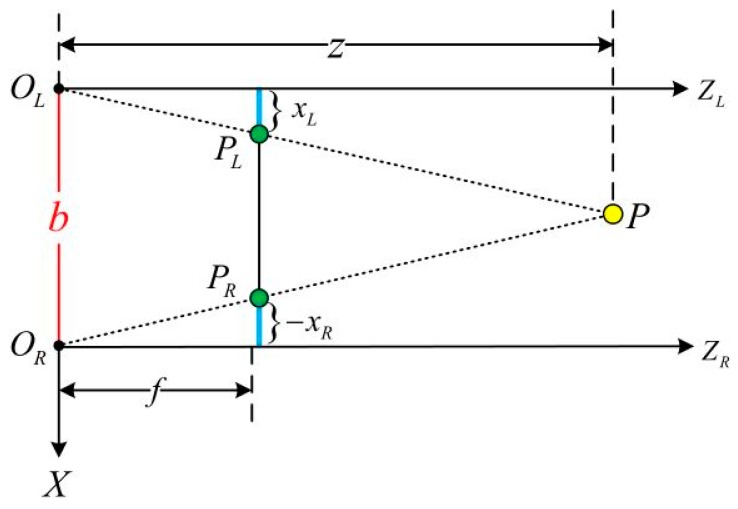
Binocular camera depth calculation schematic.

This section focuses on the principle of binocular cameras restoring scene depth. A binocular camera is generally composed of horizontally mounted left and right cameras, and the optical axes of the two cameras are parallel to each other. Ideally, the imaging model of both cameras can be regarded as a pinhole imaging model. The distance between the two cameras’ optical centers, *O_L_* and *O_R_*, is called the baseline of the binocular camera (Baseline, denoted as *b*), and the focal length parameters of the cameras are the same. As shown in Figure 3 below, there is a point *P* in space, and the imaging points of the left and right cameras are *P_L_* and *P_R_*, respectively. Since the two cameras are only displaced in the horizontal direction (X direction), the imaging points *P_L_* and *P_R_* are also different only in the X direction. We mark the coordinate of *P_L_* on the X-axis of the left-eye camera imaging plane as *x_L_* (which is a positive number), and *P_R_* is the coordinate mark of the right-eye camera imaging plane X-axis as *x_R_* (which is a negative number). According to the triangle similarity principle, $\Delta PP_{L}P_{R}$ and $\;\Delta PO_{L}O_{R}$ have similar relationships, as follows:

(10) $$\frac{z-f}{z}=\;\frac{b-x_{L}+x_{R}}{b}$$

Organize the above formula to get:

(11) $$z=\frac{fb}{d},\;d=x_{L}-x_{R}$$

In the formula, *d* represents the difference between the abscissas of the left and right image points, called Disparity. As can be seen from Equation (11), the disparity *d* is inversely proportional to the distance *z*, the larger the disparity, the smaller the measured distance. Considering the limit case, when the disparity is only one pixel size, at this time, the depth measured by the binocular camera has a theoretical maximum value *z*_max_, and *z*_max_ is finally determined by *fb*. In general, the longer the baseline of the two cameras, the farther the maximum distance the binocular camera can measure.

According to the above, if you want to calculate the distance from the space point *P* to the camera (depth *z*), you must know:

(1) Camera focal length *f* and camera baseline *b*. These two parameters can generally be obtained by camera calibration.
(2) Disparity *d*, the corresponding relationship between the imaging points x and y of the left and right cameras in the space is the core problem of the binocular visual odometer.

### 4.2. Pose Estimation

**Figure 4:**
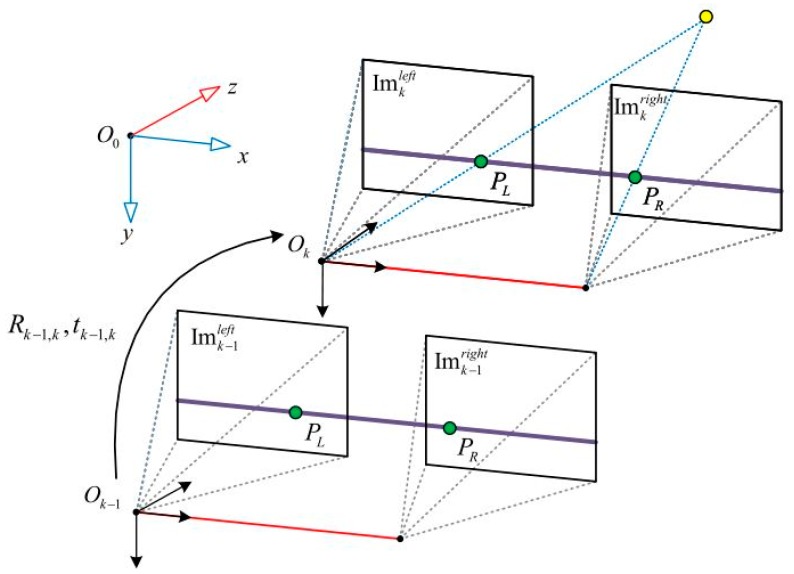
Binocular camera coordinate system diagram.

In the binocular visual odometer system, the binocular camera simultaneously acquires the left and right sequence images at each moment. The left eye image sequence is: Im0,kleft={Im0left, … ,Imkleft}, and the right eye image sequence is: Im0,kright={Im0right, … ,Imkright}. The camera coordinate system is as shown in Figure 4, in which the camera coordinate *O*_0_ whose initial time is 0 is taken as the global coordinate. The relative positional transformation of the two cameras at adjacent time *k* − 1, *k* can be represented by matrix: $T_{k-1,k}\in {\mathbb{R}}^{4\times 4}$, and the specific form is as follows:

(12) $$T_{k-1,k}=\left[\begin{array}{cc} R_{k-1,k} & t_{k-1,k}\\ 0 & 1 \end{array}\right]$$

where $R_{k-1,k}\in {\mathbb{R}}^{3\times 3}$ represents the rotation matrix of the relative positions of the two cameras, and $t_{k-1,k}\in {\mathbb{R}}^{3\times 1}$ represents the translation matrix. Assuming that the current time is *tc*, the pose *E_tc_* relative to the initial time *k* = 0 can be obtained by $T_{k-1,k}(k=1,\;\ldots \;,tc)$ cumulative transformation. The specific formula is as follows:

(13) $$E_{tc}=\overset{tc}{\underset{1}{\prod }}T_{k-1,k}$$

Feature point-based methods [25] have always been the mainstream solution in the field of visual odometers. The program runs stably, is not sensitive to illumination and dynamic targets, and is widely used in engineering fields. The feature point-based binocular visual odometer system generally includes the following steps:

Initialization:

(1) Perform feature point detection on the left and right images;
(2) Stereo matching the feature points;
(3) Triangulation method to restore depth.
(4) New left and right image sequence:
(5) Perform feature point detection and matching on the new left and right images, and calculate the depth by using the triangulation method;
(6) Feature matching of images of the previous frame and the current frame;
(7) The pose is solved using the calibrated camera parameters based on the calculated depth and the already matched feature points.

**Figure 5:**
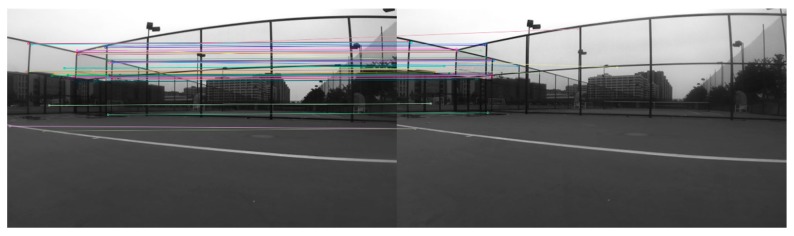
Stereo matching.

In the visual odometer, the feature points are generally extracted, tracked, matched, and screened, and the poses are estimated by the already matched feature points. Although the classic SIFT (scale-invariant feature transform) and SURF (speeded up robust features) feature point extraction algorithms are better, the calculation is very large, and the binocular camera was used in the experiment. The data processing capacity was significantly larger than that of the monocular camera. Therefore, we used the ORB (Oriented FAST and Rotated BRIEF) feature point extraction algorithm [26] with less computation and better real-time performance. In this paper, since the number of detected feature points may be very large in actual scenarios, the Fast Approximate Nearest Neighbor (FLANN) algorithm [27] was used to match feature points and improve real-time performance. The images of the experimental scene taken by the Zed camera were extracted and matched by the feature points, as shown in Figure 5. In general, visual-based odometer systems will experience cumulative drift and will not provide long-term reliable positioning for indoor vehicles. This is because the visual odometer generally estimates only the relative motion relationship between the current frame and the previous frame image. The position, velocity, and attitude errors calculated at the current time will be passed to the next moment. After a period of time, the estimated trajectory will no longer be accurate.

## 5. Data Fusion Strategy

According to the above content, due to multipath effects, non-line of sight (NLOS) and other factors, the UWB system alone cannot provide stable, reliable, and accurate positioning information for indoor mobile vehicles. The binocular visual odometer can obtain the 6-degree-of-freedom movement (position, attitude) of the camera, and obtain the relative positioning information of the moving vehicle. However, the positioning error will accumulate over time and cannot provide long-term reliable positioning for indoor moving vehicles. In order to overcome the shortcomings of the above two positioning schemes, this paper intends to integrate UWB and the binocular visual odometer. After fusing, UWB can correct the cumulative error produced by the binocular visual odometer, while the binocular visual odometer can also smooth the UWB measurement data and compensate for the deficiencies caused by multipath.

### 5.1. System Structure Design

Referring to the fusion scheme of UWB and other sensors, a tightly coupled or loosely coupled approach can be used. In the loose coupling method, the UWB raw distance measurement is first obtained by triangulation or least squares to obtain the position estimate, then, the UWB position estimate is used as data to integrate with other sensors [28]. In contrast to loose coupling, the tight coupling method directly uses the original TOA measurement for each anchor point [15,29]. Since the loose coupling method requires pre-processing of the UWB raw measurement data, in some cases, the UWB information is lost. With a tightly coupled approach, we can make the most of the available information for UWB. Therefore, for UWB and the binocular visual odometer, this paper adopts the tight coupling method.

Sensor information fusion methods mainly include: weighted average method, Kalman filter, Bayesian reasoning, neural network, and other algorithms. Although the weighted average method is simple and intuitive, it is difficult to obtain an optimal weighted average, and it takes a lot of time to calculate the optimal weighted average. The information in Bayesian inference in multi-sensor fusion is described as probability distribution, which requires prior probability and likelihood function, and the analysis and calculation is very complicated. The neural network algorithm trains and adjusts the weight of the network according to the input data samples, however, the neural network requires a large amount of data and has poor real-time performance. 

The Kalman filter generally uses a linear system state equation, combined with other input information and observation data, to optimally estimate the state of the system. The Kalman filter requires the estimated value of the system state at the previous moment and the observation information at the current time to estimate the optimal value of the current state. Because the Kalman filter is easy to program and can process and update the data collected in the field in real time, it is widely used in the engineering field. 

The system model and the measurement model of the fusion algorithm in this paper can be determined, and the system model is linear, so we use the linear Kalman filter. Since the system model and noise characteristics have an impact on the performance of the Kalman filter, in practical applications, the statistical properties of the noise are difficult to obtain. Based on the above, this paper uses the Adaptive Kalman Filter, which can dynamically estimate the system noise covariance matrix *Q* and the observed noise covariance matrix *R*.

**Figure 6:**
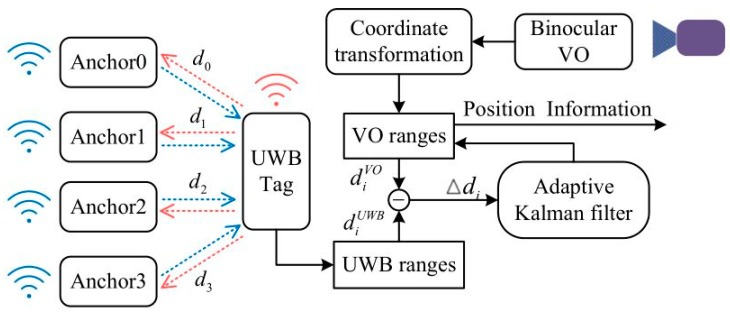
Data fusion system diagram.

The UWB/binocular VO data fusion system structure designed in this paper is shown in Figure 6. The data information of the UWB system is the original distance measurement $d_{i}^{UWB}(i=0,1,2,3)$, and the measurement information is the TOF distance from the UWB tag to the four anchor points. However, the feature-based binocular visual odometer can obtain the relative position with depth information, but it cannot output the distance of four anchor points like the UWB system, and needs further processing. The relative position of the binocular VO system solution is converted into a distance measurement similar to the UWB system, which generally requires two steps. Step 1: since the binocular VO obtains the relative position information of the carrier, it needs to be converted into global position coordinates. Step 2: Calculate the Euclidean distance of the binocular VO global position coordinates to the four anchor points based on the known x, y, z direction coordinates of the anchors. The Euclidean distance $d_{i}^{VO}(i=0,1,2,3)$ finally obtained is the distance measurement of the binocular VO.

According to the illustrated fusion system structure, after obtaining the distances of UWB and binocular VO respectively, the error amount $\Delta d_{i}$ of $d_{i}^{UWB}$ and $d_{i}^{VO}$, is used as the measurement input of the Adaptive Kalman Filter. $\Delta d_{i}$ represents the optimal state estimate after filtering, which will feed back the distance measurement of the binocular VO. The corrected binocular VO distance $d_{i}^{VO}$ is solved by least squares, and the final global position of the fusion system can be obtained.

### 5.2. Filtering Algorithm Model

In the Kalman filter used in this paper, the system state equation is as follows:

(14) $$x_{k}=Ax_{k-1}+w_{k}$$

where, $x_{k}$ represents the system state vector of the fusion system at time *k*, and *A* represents the state transition matrix from time k−1 to time *k*. $w_{k}$ represents system noise, and the noise satisfies the Gaussian white noise $w_{k}\sim N$*(0*,*Q).* The specific definition of $x_{k}$ is as follows, representing the error of the carrier tag to the distance of four anchor points, and the state transition matrix *A* is the identity matrix *diag*(1,1,1,1).

(15) $$x_{k}={\left[\Delta d_{0}\;\Delta d_{1}\;\Delta d_{2}\;\Delta d_{3}\right]}^{T}\;$$

The measurement equation for the fusion system is:

(16) $$z_{k}=Hx_{k}+v_{k}$$

where, $z_{k}$ is the observation vector of the fusion system at time *k*, and H is the observation matrix. $v_{k}$ represents observation noise, and the noise satisfies the Gaussian white noise $v_{k}\sim N$*(0*,*R)*. The specific definition of $z_{k}$ is as follows, representing the difference between the distance $d_{i}^{VO}$ of the binocular VO solution and the TOF distance $d_{i}^{UWB}$ of the UWB system. The observation matrix H is an identity matrix *diag*(1,1,1,1).

(17) $$z_{k}={\left[d_{0}^{VO}-d_{0}^{UWB\;}\;d_{1}^{VO}-d_{1}^{UWB\;}\;d_{2}^{VO}-d_{2}^{UWB\;}\;d_{3}^{VO}-d_{3}^{UWB}\right]}^{T}$$

According to the variable parameters defined above, the complete adaptive Kalman filter prediction process is as follows. Where ${\hat{x}}_{k-1}$ represents the optimal estimate of the state at time k−1, and ${\hat{x}}_{k,k-1}$ represents the predicted value of the state at time k obtained from the system state equation. *P*_*k*−1_ represents the covariance matrix of the error between the updated value of the state and the true value at time k−1. *P_k_*,_*k*−1_ represents the covariance matrix of the error between the predicted value and the true value of the state at time k.

(18) $${\hat{x}}_{k,k-1}=A{\hat{x}}_{k-1}$$

(19) $$v_{k}=z_{k}-H{\hat{x}}_{k,k-1}$$

(20) $${\hat{V}}_{k}=\frac{1}{k}\sum_{i=1}^{k}v_{i}v_{i}{}^{T}$$

(21) $$Q_{k}=K_{k-1}{\hat{V}}_{k}K_{k-1}{}^{T}$$

(22) $$P_{k,k-1}=AP_{k-1}A^{T}+Q_{k}$$

The complete adaptive Kalman filter update process is as follows, where *K_k_* represents the Kalman gain matrix, *K_k_* represents the optimal estimate of the state at time *k*, and *P_k_* represents the covariance matrix of the error between the updated and the true value at time *k*. In the iterative process, the system noise covariance matrix *Q_k_* and the observed noise covariance matrix *R_k_* are dynamically updated.

(23) $$R_{k}={\hat{V}}_{k}-HP_{k,k-1}H^{T}$$

(24) $$K_{k}=P_{k,k-1}H^{T}{\left[HP_{k,k-1}H^{T}+R_{k}\right]}^{-1}$$

(25) $${\hat{x}}_{k}={\hat{x}}_{k,k-1}+K_{k}v_{k}$$

(26) $$P_{k}=(I-K_{k}H)P_{k,k-1}$$

## 6. Experimental Study and Analysis of Results

In this section, two experiments were performed to verify the performance of the proposed data fusion algorithm. First, we introduce the hardware configuration of the experiment, then, introduce the experimental scenarios and steps. Finally, the experimental results are presented and discussed. The experimental results show that the proposed binocular VO/UWB data fusion algorithm is superior to the UWB positioning system alone.

### 6.1. Hardware Setup

**Figure 7:**
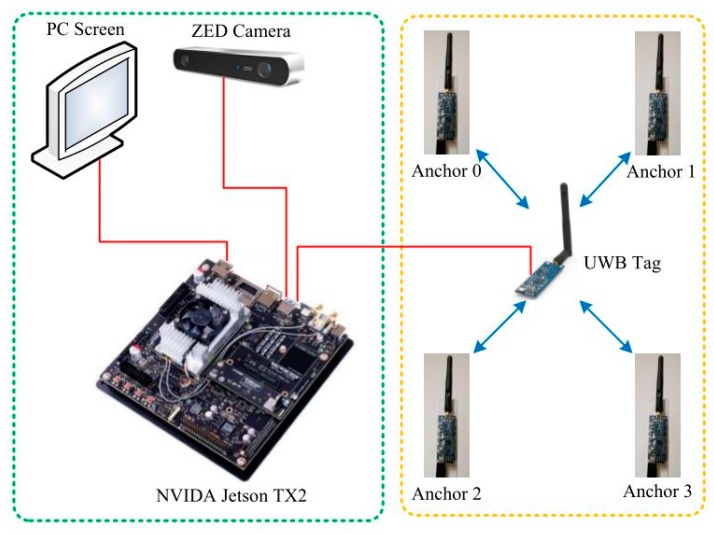
Hardware setup.

The hardware platform used in this article is the NVIDA Jetson TX2 embedded development board. The TX2 is a modular AI (artificial intelligence) supercomputer with NVIDA PascalTM architecture. The TX2 is small, but powerful, energy efficient, and ideal for smart edge devices such as robots, drones, smart cameras, and portable medical devices. The TX2 used in this article integrates the Ubuntu16.04 system. The hardware settings are shown in Figure 7, and one end of the TX2 is connected to a peripheral, such as a display.

The UWB hardware used in the experiment is equipped with the DW1000 module. The module uses the STM32F103T8U6 MCU as the main control chip, including USB and serial port. The module can be used as a base station or as a tag to switch via USB commands. The theoretical positioning accuracy of the UWB system is about 15 cm, and the communication range is about 300 m. The binocular camera used was a ZED stereo camera manufactured by Stereolabs, which can acquire the binocular RGB image simultaneously and then calculate the depth on the computer by the binocular principle. The detected maximum depth distance can reach 20 m. This article uses the Robotic Operating System (ROS) [30] to connect components, because it provides a standard set of ROS interfaces and related communications.

### 6.2. Analysis of Results

**Figure 8:**
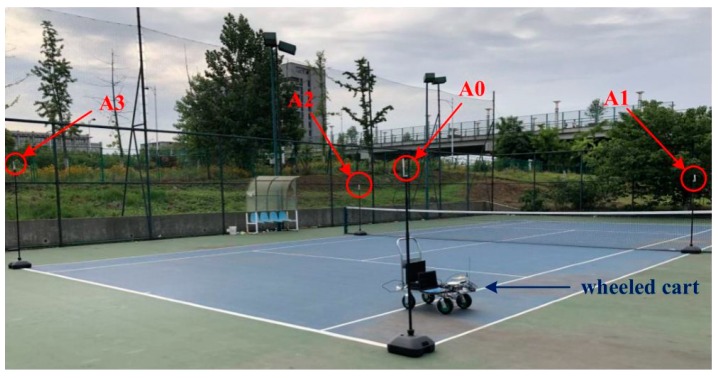
Experimental scene.

The experimental hardware platform was securely mounted on a wheeled cart that traveled along a defined route. In order to obtain a real trajectory of driving, the experiment was carried out in a scene equipped with an RTK (real-time kinematic) system. As shown in Figure 8, the experimental scene was a rectangular open space with a length of about 11.6 m and a width of about 10.9 m. In order to get an accurate *Z*-axis position, the experiment needed to set 4 anchor points (A0–A3). Among them, the height of A3 placement was preferably 1 m or 0.5 m higher than A0/A1/A2, and A0/A1/A2 were in the same plane. The position parameters of each anchor point are shown in Table 1. The experiment was performed twice in total, the trajectory of the first experiment was an approximately square closed curve, and the second trajectory was an S-shaped curve.

**Figure 9:**
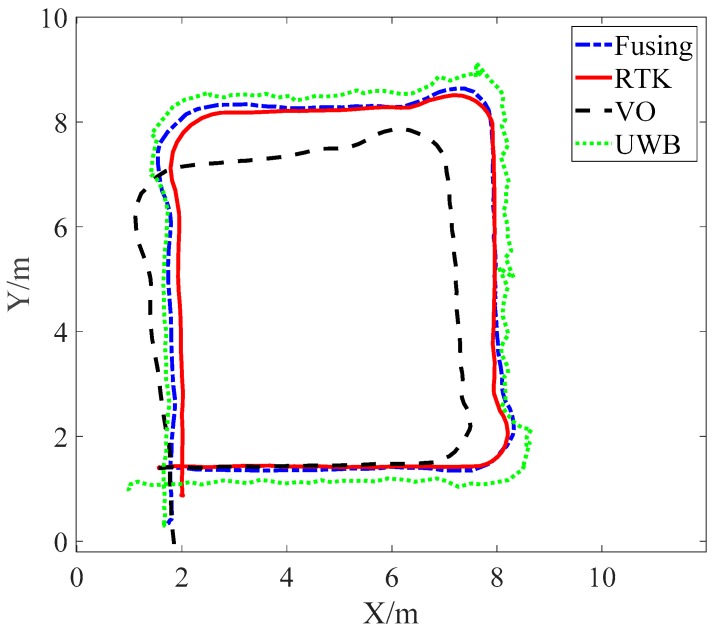
The comparison of trajectories in the X–Y plane.

In the first test, a wheeled cart equipped with a hardware platform produced an approximately square closed curve. The original information such as the image sequence and UWB distance measurement collected by the experiment was processed offline. According to the contents of Section 3, Section 4 and Section 5, the UWB, binocular VO, and the merged driving path are compared as shown in Figure 9. Since the wheeled cart was primarily traveling on a flat surface, Figure 9 shows only the paths along the X and Y axes for ease of comparison. According to Figure 9, the actual path is obtained by the RTK system and drawn with red lines as a reference. The other three paths are calculated using UWB, binocular VO, and the Adaptive Kalman fusion algorithm.

As shown in Figure 9, the path obtained by the fusion algorithm proposed in this paper is closer to the actual path than the path obtained by UWB and binocular VO independently. According to the UWB system positioning principle, it can obtain the absolute positioning information of the vehicle. The UWB positioning error does not diverge compared to the actual path. Although the depth of binocular VO can be obtained by the binocular principle, it is not accurate enough and is smaller than the actual depth information. At the same time, the trajectory of the binocular VO has a large drift, the positioning error accumulates over time and cannot work alone for a long time. Comparing the driving path, we can qualitatively analyze that the UWB/VO fusion algorithm proposed in this paper not only has better positioning accuracy than single UWB positioning and binocular VO positioning, but also smooths the UWB driving path.

**Figure 10:**
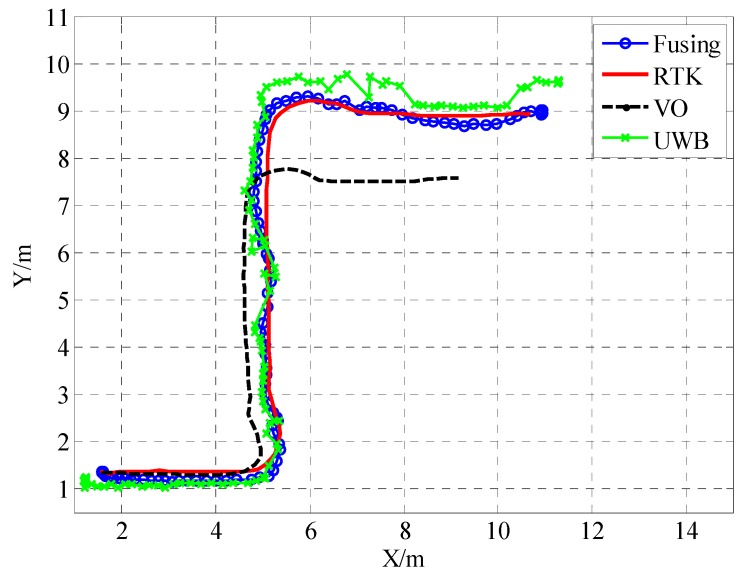
The comparison of trajectories in the X–Y plane.

In the second test, the wheeled cart produced an S-shaped curve. Figure 10 shows the path of the S-shaped curve along the X and Y axes. As shown, the RTK trace represents the actual path and is drawn with a red line as a reference. The other three paths represent the UWB, binocular VO, and adaptive Kalman fusion algorithms, respectively. The UWB positioning error does not accumulate compared to the actual path and the UWB path is closer to the actual path than the binocular VO path. The depth information obtained by the binocular principle is smaller than the actual depth. At the same time, the path of the binocular VO has cumulative drift, and the positioning accuracy decreases with time. It is impossible to work alone for a long time. As shown in the second test results, the UWB/VO tight coupling algorithm proposed in this paper is not only closer to the actual path than the other two positioning methods, it can also smooth the UWB driving path and eliminate the glitch generated by UWB.

**Figure 11:**
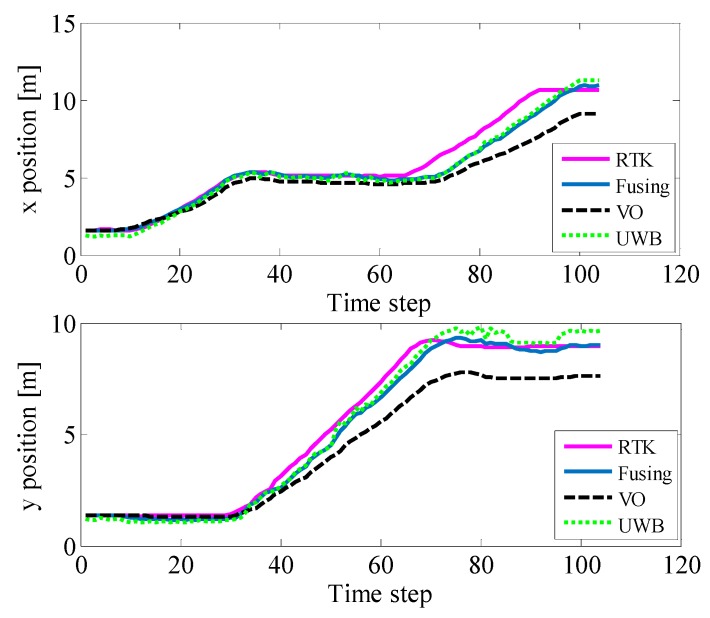
Position of different positioning methods in two directions.

Figure 11 represents the positional information of the UWB, binocular VO, and adaptive Kalman fusion algorithms on the x and y axes, respectively. As shown in the figure, although the position of the binocular VO is close to the actual position at the beginning, it gets worse and worse during the running because of the error accumulation. Although UWB did not perform well at the beginning, it did not drift and the overall positioning performance was stable. The fusion algorithm is better than the other two in the beginning, the driving process, and the ending phase. At the same time, the location of the fusion algorithm is smoother than UWB.

**Figure 12:**
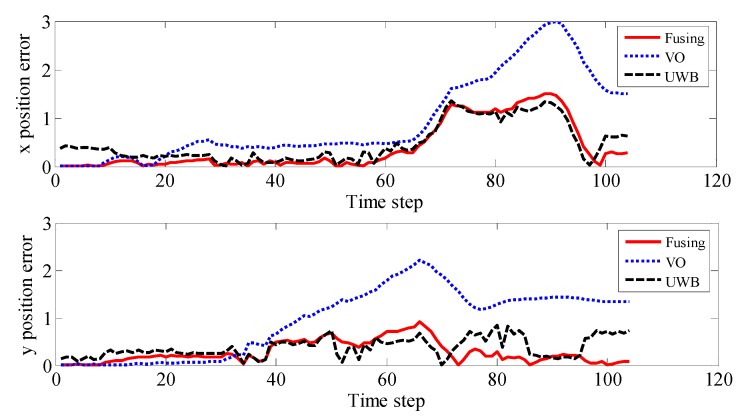
Position error of different positioning methods in X and Y directions.

Figure 12 shows the absolute position error of the UWB, binocular VO, and adaptive Kalman fusion algorithms along the x and y axes. As shown in the figure, it can be observed that due to the drift of binocular VO, the position error is significantly higher in the x and y directions than the other two methods. The position error of a single UWB is between the other two methods. The UWB/binocular VO fusion algorithm has less positional error than the other two in the beginning, driving, and ending phases. In summary, the UWB/VO tight coupling algorithm proposed in this paper can not only improve the positioning accuracy, but also smooth the UWB driving path and eliminate the glitch generated by UWB. At the same time, it can also suppress the drift of the binocular VO to improve the overall positioning performance.

**Figure 13:**
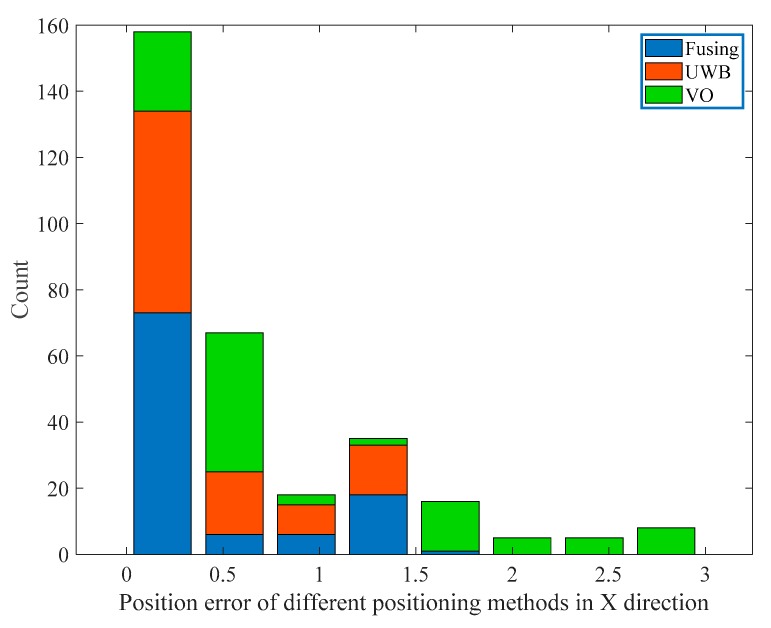
Position error distribution histogram in the X direction.

**Figure 14:**
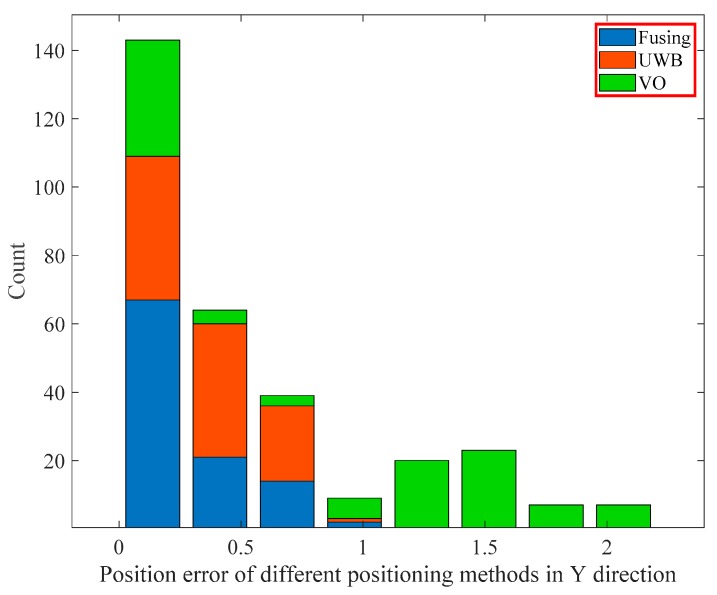
Position error distribution histogram in the Y direction.

We did a statistical analysis of the position error of UWB, binocular VO, and fusion algorithm, and divided the position error of the three positioning methods along the x and y axes into eight intervals according to the order from small to large and then calculated the number of location points in each interval. Finally, we plotted the stack histogram of the position error distribution. Figure 13 and Figure 14 show the position error distribution of the three positioning methods in the x and y directions, respectively. It can be observed that the location points of the fusion algorithm are mainly distributed in the first interval which represents the smallest position error, and in the first interval, the number of the location points of the fusion algorithm are significantly more than the other two positioning methods. Moreover, the location points of the fusion algorithm and the single UWB positioning method are mainly distributed in the first three intervals, while the single VO method is still distributed in other intervals due to the cumulative error.

According to the histogram distribution, we calculated the percentage of the number of location points in the first three intervals of the three positioning methods. As shown in Table 2, in the x direction, the percentage of the number of location points of the fusion algorithm in the interval (0, 0.3729) was 70.20%, as shown in Table 3, in the y direction, the percentage of the fusion algorithm in the interval (0, 0.2757) was 64.42%, they are significantly larger than the other two positioning methods. The above statistical analysis has also verified that the UWB/binocular VO fusion algorithm proposed in this paper can effectively improve the positioning accuracy.

The error is analyzed by the concept of Cumulative Distribution Function (CDF). According to Equation (27), the error distribution of the three positioning methods can be obtained by:

(27) F(x)=P(X≤x)

**Figure 15:**
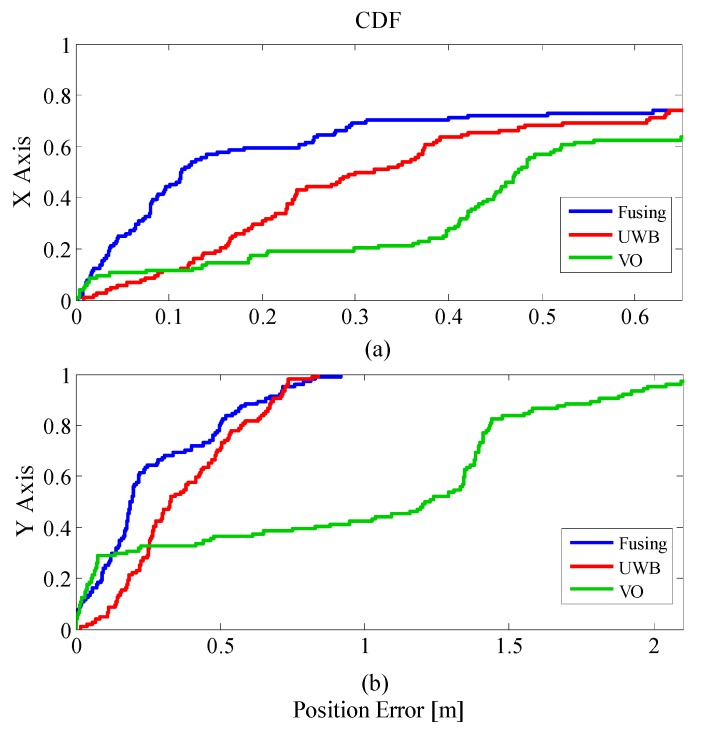
Position error, cumulative distribution function.

As shown in Figure 15, in addition to the VO positioning effect being better at the beginning stage, in other stages, the positioning error of the fusion algorithm was significantly smaller than the other two positioning methods in both the X direction and the Y direction. This also verifies that in the indoor environment, the positioning scheme of the fusion algorithm can effectively improve the positioning effect.

As shown in Table 4, the average errors along the x and y axes of the UWB, binocular VO, and fusion algorithms are listed separately. It can be observed that the average error of the fusion algorithm was smaller than a single UWB and a single VO positioning method. Experiments show that compared to a single UWB positioning system, the proposed fusion algorithm can improve the positioning accuracy by 16% on the x-axis and 27% on the y-axis.

## 7. Conclusions

Because a single UWB positioning system is affected by factors such as non-line of sight and multipath, it cannot meet the high precision requirements of vehicle interior positioning. This paper proposed a sensor fusion scheme based on binocular VO (visual odometer)/UWB (ultra-wideband). Firstly, the structural design of the fusion system was introduced. Then the fusion algorithm based on the adaptive Kalman filter was elaborated. Finally, the experimental data of two sets of wheeled carts were collected to verify the feasibility of the algorithm. The experiments show that the binocular VO/UWB fusion method can significantly improve the positioning accuracy of mobile vehicles in indoor environments compared to a single UWB positioning scheme and feature point-based binocular visual mileage calculation. The proposed binocular VO/UWB tightly coupled localization algorithm is of great significance to solve the problem of indoor positioning of mobile vehicles under the condition that GPS cannot work normally.

## Figures and Tables

**Table 1:** The global location information of anchors.

	X (m)	Y (m)	Z (m)
Anchor 0	0	0	2.0
Anchor 1	11.6	0	2.0
Anchor 2	11.6	10.9	2.0
Anchor 3	0′	10.9	2.5

**Table 2:** Percentage of location points in the x direction.

Position Error (m)	Fusing	UWB Only (Ultra-Wideband)	VO Only (Visual Odometer)
(0, 0.3729)	70.20%	58.65%	23.08%
(0.3729, 0.7459)	5.77%	18.27%	40.38%
(0.7459, 1.1188)	5.77%	8.65%	2.88%

**Table 3:** Percentage of location points in the y direction.

Position Error (m)	Fusing	UWB Only (Ultra-Wideband)	VO Only (Visual Odometer)
(0, 0.2757)	64.42%	40.38%	32.69%
(0.2757, 0.5514)	20.19%	37.50%	3.85%
(0.5514, 0.8271)	13.46%	21.15%	2.88%

**Table 4:** Mean error of three positioning methods.

Mean Error	Fusing	UWB Only	VO Only
X (m)	0.3993	0.4752	0.9405
Y (m)	0.2769	0.3786	0.9357
